# Study on the Effect of Natural Aging and PAV Aging on Asphalt Binder Based on Rheology and Microstructural Composition

**DOI:** 10.3390/ma18225096

**Published:** 2025-11-10

**Authors:** Fujin Hou, Yunding Zhu, Meng Guo, Wenwu Zhang, Bolaxiake Hailati

**Affiliations:** 1 Shandong Hi-Speed Group Co., Ltd. Expressway Operation Center, Jinan 250098, China; 2 State Key Laboratory of Bridge Engineering Safety and Resilience, Beijing University of Technology, Beijing 100124, China; 3 The Key Laboratory of Urban Security and Disaster Engineering of Ministry of Education, Beijing University of Technology, Beijing 100124, China; 4 Tacheng Municipal Bureau of Housing and Urban-Rural Development, Tacheng 834700, China; 15109062172@163.com

**Keywords:** natural aging, asphalt binder, aging behavior, key differences

## Abstract

Laboratory-simulated aging fails to fully replicate the complex aging behavior of asphalt binder under actual environmental conditions. This study aims to preliminarily investigate and analyze the differences between natural aging and PAV aging of asphalt binder. To achieve this objective, the asphalt binder was aged using three distinct methods: PAV aging, natural thermal-oxidative aging, and all-weather aging. The divergence in asphalt binder aging behavior was systematically investigated through encompassing low-temperature performance, chemical structure, elemental composition, molecular weight, and macroscopic and microscopic performance correlation analyses. Key findings include: the harsh environment in the cold and arid regions resulted in inferior low-temperature performance of asphalt binder after 12 months of natural thermal-oxidative and all-weather aging compared to PAV-aged asphalt binder, with the stiffness modulus at −12 °C increasing by 114.8% and 105.3%, respectively. Natural aging induced more significant asphalt binder’s chemical structural changes than PAV aging but exhibited less prominent oxidative reactions and macromolecular structure formation. Whether from a macroscopic or microscopic perspective, thermal-oxidative conditions were identified as the primary driver behind both the natural aging behavior and the aging pathway of asphalt binder. The influence of other factors on the aging behavior of asphalt binder was not significant. The poor correlation (R^2^ < 0.62) between oxygen content, molecular weight, and low-temperature performance across different aging modes underscores a fundamental divergence in aging pathways between PAV and natural aging. This study preliminarily identifies the key differences between laboratory-accelerated aging and natural aging of asphalt binder and paves the way for optimizing the parameters of laboratory-accelerated aging protocols.

## 1. Introduction

Flexible pavement has been widely adopted in high-grade highways worldwide due to its advantages, such as enhanced driving safety and reduced noise pollution. During prolonged service exposure, flexible pavement inevitably undergoes degradation caused by environmental factors including elevated temperatures, oxygen, and solar radiation. This progressive deterioration of macroscopic performance under sustained environmental exposure is defined as flexible pavement aging [1,2,3,4,5]. Extensive investigations have demonstrated that aging induces bitumen hardening and embrittlement, accompanied by significant reductions in viscoelastic performance and crack resistance, ultimately leading to premature deterioration of pavement durability [6,7,8].

Considering the prolonged aging process of asphalt during actual service, researchers have consistently sought to simulate field aging in laboratory settings to expedite sample acquisition. Conventional methods include the Thin Film Oven Test (TFOT) and Rolling Thin Film Oven Test (RTFOT) to simulate short-term aging during production, mixing, and paving stages, while the Pressurized Aging Vessel (PAV) method replicates long-term field aging through high-temperature and high-pressure conditions. In recent years, numerous studies have employed these laboratory-based accelerated aging protocols, establishing standardized procedures for asphalt aging research. However, significant discrepancies persist between the continuous high temperature/pressure conditions of simulated aging and actual pavement aging mechanisms. Under high ambient temperatures, asphalt pavement surfaces exhibit significantly elevated temperatures compared to the surrounding air. Guan et al. reported that in most Chinese cities, summer atmospheric temperatures ranging between 30 and 40 °C typically result in pavement temperatures not exceeding 70 °C [9]. This shows that there is a large thermal difference between field conditions and laboratory simulations, as standardized PAV aging protocols employ sustained 100 °C exposure—representing a 30 °C+ temperature differential from actual pavement thermal regimes. Furthermore, PAV aging fails to consider the dynamic temperature variations inherent in natural environments due to diurnal and seasonal cycles. More critically, this accelerated aging protocol, which focuses solely on elevated temperature and pressure conditions, neglects the synergistic effects of multiple environmental factors encountered during actual pavement service. In fact, as early as 1961, Traxler discovered that light could accelerate the aging of asphalt binder [10]. Liu et al. revealed that during the ultraviolet (UV)-induced aging process of asphalt binder, molecular excitation and chemical bond cleavage occur. Under these effects, unstable chemical bonds within asphalt reach an excited state or fracture, thereby becoming more prone to oxidation reactions [11]. Zhao et al. further investigated the role of water in asphalt binder aging, demonstrating that water exhibits a certain inhibitory effect on UV-induced asphalt aging [12]. Additionally, some researchers have explored the impacts of unique environmental factors on asphalt binder aging, such as acid rain and saline water [13,14,15]. It is evident that compared to PAV aging, the actual natural environmental aging factors are more complex.

While comparative assessment of PAV aging and natural aging can be conducted through macroscopic performance degradation, asphalt binder’s inherent complexity as a complex organic compound comprising approximately 300–2000 distinct chemical constituents renders the microstructural aging processes exceedingly intricate [16]. Recent advancements in microscale aging characterization methodologies have enabled researchers to systematically investigate the evolutionary behavior of asphalt binder’s internal microstructural composition during aging processes. This provided a powerful means to reveal the deep reason for asphalt binder macroscopic performance decline. Nuclear Magnetic Resonance (NMR) spectroscopy has proven effective in identifying chemical structural change during aging, including substitution, isomerization, fragmentation, association, polymerization, condensation, and cyclization [17,18,19]. Experimental investigations by Zhang, Di et al. have revealed that aging generates substitution reactions at hydrogen sites on aromatic rings, accompanied by isomerization of specific hydrocarbon types [20,21,22]. The dehydrogenation process generates free radicals that exhibit high reactivity with atmospheric oxygen, forming peroxy radicals. These reactive species abstract hydrogen atoms from adjacent asphalt binder molecules, thereby leading to radical chain reactions that ultimately lead to the formation of oxygen-containing functional groups [23,24,25]. Elemental analysis provides direct empirical evidence for characterizing asphalt binder oxidative aging, with measurable increases in oxygen content being quantitatively verified during aging processes [26,27]. Molecular-scale investigations employing Gel Permeation Chromatography (GPC) have revealed that asphalt binder aging significantly increases both average molecular weight and molecular dispersity [28,29]. These molecular-level changes amplify intermolecular friction forces within the asphalt binder, constraining molecular mobility and ultimately leading to deterioration in macroscopic performance. It is evident that aging induces evolutionary changes in the asphalt binder’s microscale structure, which consequently affects its macroscopic performance.

A substantial body of research has compared the differences between naturally aged and pressure aging vessel (PAV) aged asphalt binder from both macro- and micro-scale perspectives. However, to develop more rational laboratory-accelerated aging methods, it is essential to identify the cross-scale behavior of asphalt binder under laboratory and natural aging conditions—that is, the divergence in aging pathways induced by these two distinct aging modes. Zhang et al. reported discrepancies in rheological properties and microstructural characteristics between laboratory-accelerated aged and naturally aged asphalt [30]. Based on FTIR and rheological tests, Khalighi et al. further established a novel aging protocol, showing that treatment with 33% H_2_O_2_ at 85 °C for 3 h or at 60 °C for 6–9 h most closely replicates field-aged asphalt binder in terms of chemical and rheological properties [31]. Although some studies have addressed the distinctions between laboratory-accelerated and natural aging of asphalt binder, two critical issues remain unresolved: 1. Differences in macro–micro cross-scale behavior between laboratory-accelerated and natural aging across varying micro-scale indicators; 2. The key factors driving these divergent cross-scale behaviors between the two aging modes.

**Figure 1:**
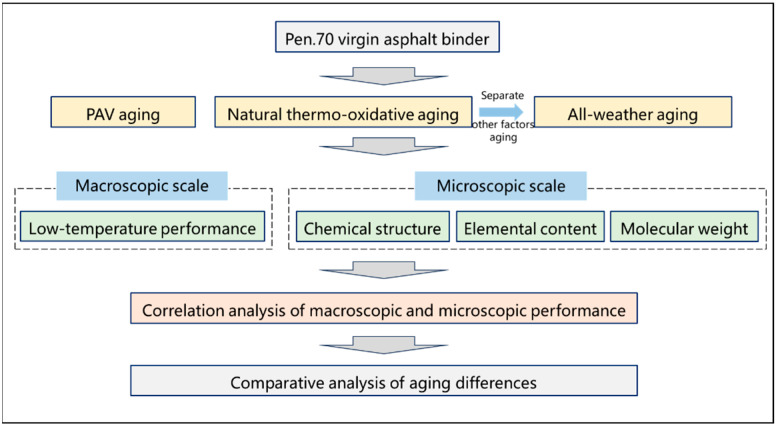
The technological roadmap.

This study aims to preliminarily clarify the differences between natural aging and PAV aging behaviors of asphalt binder. Four aging styles were implemented: short-term aging, PAV aging, 12-month natural thermal-oxidative aging, and 12-month all-weather aging. The effects of these protocols on asphalt binder’s low-temperature performance degradation, chemical structure evolution, elemental composition variation, and molecular weight distribution were systematically analyzed. Furthermore, macroscopic and microscopic performance correlation analyses revealed critical discrepancies between PAV aging and natural aging behaviors. These findings provide a theoretical foundation for calibrating deviations between laboratory-simulated aging and natural aging behaviors of asphalt binder, and for achieving accurate simulation of natural aging through controlled laboratory-simulated aging. The technological roadmap for the paper is shown in Figure 1.

## 2. Materials and Methods

### 2.1. Materials

The experiment employed Pen.90 virgin asphalt binder, with its fundamental technical indexes detailed in Table 1. The standard test methods of bitumen and bituminous mixtures for highway engineering are found in JTG E20-2011 [32].

### 2.2. Test Methods

#### 2.2.1. Laboratory-Simulated Aging Methods

**Figure 2:**
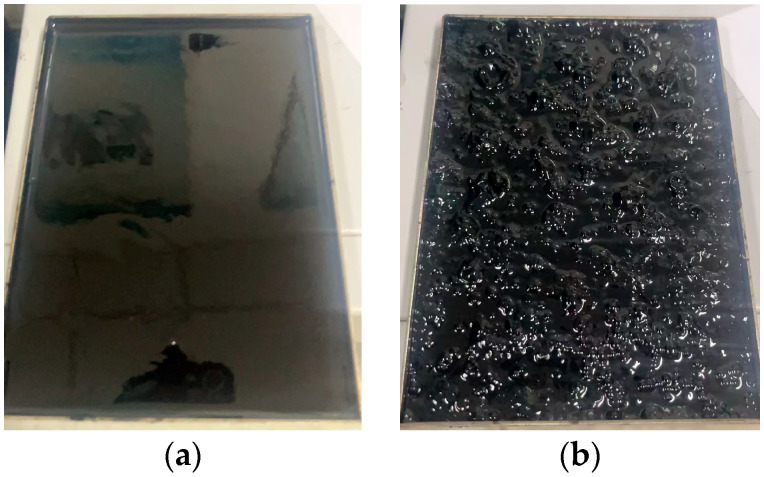
Asphalt binder samples: (**a**) Asphalt binder samples aged by the Thin Film Oven Test (**b**) Asphalt binder samples aged by the Pressure Aging Vessel.

The asphalt binder was placed in a thin-film oven for aging. The aging temperature was set to 163 °C, and the asphalt binder was aged for 5 h to obtain short-term aged asphalt binder. Subsequently, the short-term aged asphalt binder was transferred to a pressure aging vessel (PAV), where it underwent further aging at 100 °C under 2.1 MPa pressure for 20 h, resulting in long-term aged asphalt binder. The asphalt binder samples aged by the Thin Film Oven Test (TFOT) and the Pressure Aging Vessel (PAV) are shown in Figure 2.

#### 2.2.2. Natural Aging Methods

The natural aging of asphalt binder samples was conducted at the Field Scientific Observation and Research Station in the Cold-Arid Regions of Northwest China, located in Dunhuang City, Gansu Province. This region exhibits a dry climate with minimal rainfall, significant diurnal temperature fluctuations, and short, windy spring and autumn seasons. Frequent spring dust storms and intense ultraviolet radiation further define its environmental conditions. These complex climatic factors drive intricate aging behaviors in asphalt binder, severely undermining pavement durability.

**Figure 3:**
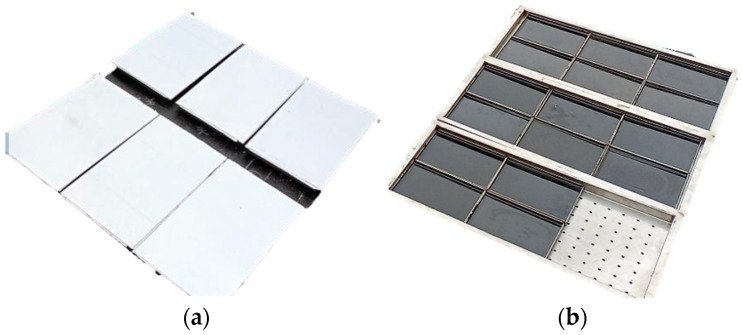
The natural aging style of asphalt binder: (**a**) Natural thermal-oxidative aging style; (**b**) All-weather aging style.

To systematically compare PAV aging with natural aging, two distinct natural aging styles were applied to short-term thin-film oven-aged asphalt samples. All aging samples were mounted on racks 0.5 m above ground level and subjected to a 12-month aging period under field conditions. By controlling the mass of the asphalt binder samples, the film thickness of all specimens was maintained at 3.2 mm, consistent with the film thickness used in laboratory aging procedures. All natural aging samples were horizontally positioned in an unobstructed open-air testing ground, ensuring full exposure to direct sunlight and maintaining a sufficient distance from structures, trees, or other objects that could cause shading or create localized microclimatic effects. This setup guaranteed that the aging process was primarily driven by regional climatic conditions. During the 12-month aging period of the asphalt binder samples, the ambient conditions featured an average temperature of 10.6 °C, 159 annual frost-free days, 3039.1 h of sunshine, 11.7 mm of precipitation, and 1051 mm of evaporation. The asphalt binder sample shown in Figure 3a was shielded by an opaque cover, restricting its exposure solely to temperature and oxygen while isolating it from light, water, dust, and other environmental factors. It was abbreviated as 12-O. This style is designated as natural thermal-oxidative aging style. The asphalt binder sample depicted in Figure 3b was fully exposed to the natural environment, subjecting it to all environmental factors including light, moisture, and dust. This style, designated as all-weather aging style, enables systematic analysis of non-thermal-oxidative factors on asphalt binder aging. It is abbreviated as 12-A.

Considering that the asphalt binder samples obtained in the field contained a significant amount of dust, they were dissolved using trichloroethylene. The solution was then filtered through 15 micrometer, 5 micrometer, and 1 micrometer membranes, respectively. Finally, the trichloroethylene was removed by rotary evaporation to obtain the asphalt binder samples for experimental testing.

#### 2.2.3. Test Methods for Low-Temperature Performance

The low-temperature performance of asphalt binder samples was evaluated using a dynamic shear rheometer (DHR-2) equipped with 4 mm aluminum parallel plates. Testing was conducted at three temperatures: −18 °C, −12 °C, and −6 °C, with an angular frequency sweep ranging from 0.2 to 100 rad/s under controlled strain mode at 0.1%.

To obtain the stiffness modulus *S* and creep rate *m* that exhibit the same mechanical response as the BBR test, the DSR data were converted. Equations (1)–(3) were applied to transform the shear modulus into tensile compliance. Finally, the stiffness modulus *S* and creep rate *m* were obtained by back-calculating from the data on the creep compliance curve. For details of the calculation methodology, readers are referred to the work of Hajj et al. [33].

(1) $$\begin{array}{c} \log\left|G\left(\omega \right)\right|=\delta +\frac{\alpha }{1+e^{\beta +\gamma \log\left(\omega \right)}} \end{array}$$

(2) $$\begin{array}{c} J\left(t\right)=J\prime \left(\omega \right)+0.4J\prime \prime \left(0.4\omega \right)-0.014J\prime \prime \left(10\omega \right) \end{array}$$

(3) $$\begin{array}{c} D\left(t\right)=\frac{J\left(t\right)}{2(1+v)} \end{array}$$

In the equations, $G\left(\omega \right)$ represents the shear modulus (kPa), *ω* represents the frequency at the reference temperature, J represents the shear compliance, $J^{\prime }$ represents the storage compliance, $J^{\prime \prime }$ represents the loss compliance, ν represents Poisson’s ratio, α, β, and *γ* represent coefficients, and D represents the tensile compliance.

#### 2.2.4. Test Methods for Chemical Structure

1. Chemical structure analysis

The experiments employed a nuclear magnetic resonance (NMR) spectrometer (ASCEND™ 400 [AVANCE HD III], Bruker Corporation, Billerica, MA, USA) with a magnetic field strength of ≥9.39 T (400 MHz) and magnetic field drift ≤ 8 Hz/h. A 10 mg asphalt binder sample was dissolved in 0.5 mL of deuterated chloroform and subjected to NMR analysis. The acquired spectra were processed using TopSpin 4.5.0 software to quantify assigned attributed hydrogen content, thereby characterizing the chemical structure of the asphalt binder.

2. Elemental Content Analysis

The experiments were performed using a Vario EL cube elemental analyzer (Elementar Analysensysteme GmbH, Langenselbold, Germany) employing the micro-combustion method. Samples were encapsulated in tin containers and combusted at 1200 °C to achieve complete decomposition. The resultant products were transported via helium carrier gas to the separation and detection unit, where they underwent adsorption–desorption processes for chromatographic separation.

3. Average Molecular Weight Analysis

The experiments utilized a gel permeation chromatography (GPC) system (Waters 515-717-2410, Waters, Milford, Massachusetts, USA) equipped with a refractive index detector (Waters 2410) and a column set comprising three Waters Styragel columns (HT6E-HT5-HT3) connected in series. The column temperature was maintained at 35 °C, and the elution flow rate was set to 1 mL/min. Calibration was performed using polystyrene (PS) standards with peak molecular weights ranging from 1.62 to 2.3 million, employing a 14-point calibration curve constructed from the PS standards. Asphalt samples were prepared at concentrations of 10–15 mg/mL in tetrahydrofuran (THF) as the mobile phase, with an elution flow rate of 1 mL/min. The number-average molecular weight ($M_{n}$), weight-average molecular weight ($M_{w}$), and polydispersity index (D) were calculated using Equations (4)–(6).

(4) $$\begin{array}{c} M_{n}=\frac{\sum N_{i}M_{i}}{\sum N_{i}} \end{array}$$

(5) $$\begin{array}{c} M_{w}=\frac{\sum N_{i}M_{i}^{2}}{\sum N_{i}M_{i}} \end{array}$$

(6) $$\begin{array}{c} D=\frac{M_{w}}{M_{n}} \end{array}$$

In the equations, $M_{n}$ represents the number-average molecular weight, $M_{w}$ represents the weight-average molecular weight, D represents the polydispersity index, and $N_{i}$ represents the number of molecules with a molecular weight of $M_{i}$.

## 3. Results and Discussion

### 3.1. The Influence of Different Aging Styles on the Low-Temperature Performance of Asphalt Binder

**Figure 4:**
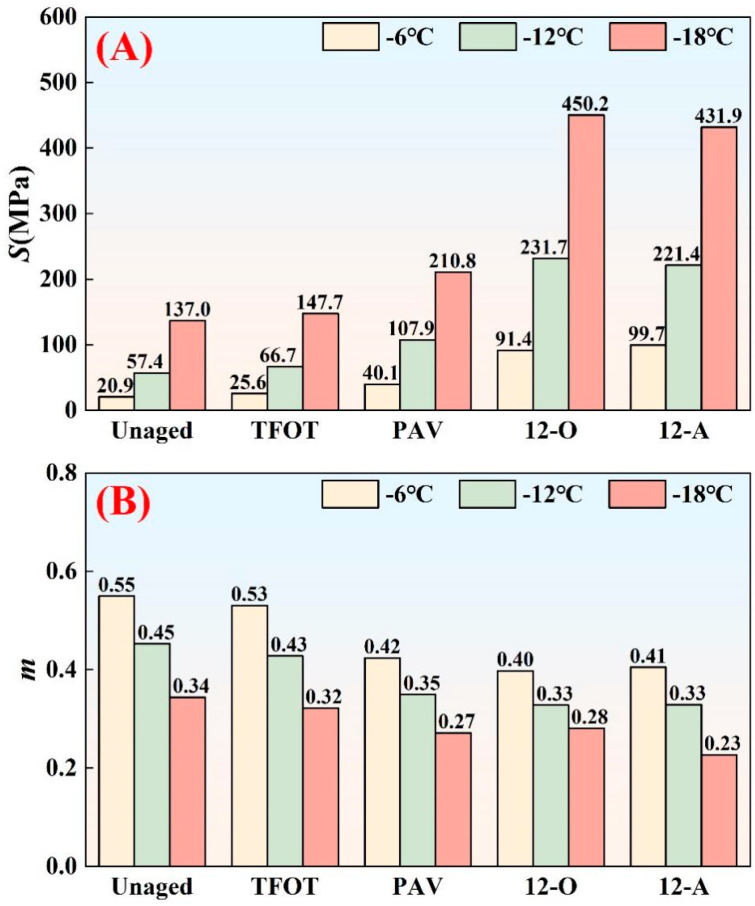
The stiffness modulus and creep rate of asphalt binder under different aging styles: (**A**) stiffness modulus; (**B**) creep rate.

As a temperature-sensitive material, asphalt binder exhibits viscoelastic properties that are significantly influenced by temperature. At low temperatures, its elastic characteristics become more pronounced, thereby increasing susceptibility to cracking [34]. The U.S. Strategic Highway Research Program (SHRP) introduced stiffness modulus (*S*) and creep rate (*m*) as key parameters for evaluating asphalt binder’s low-temperature performance. The measured stiffness modulus and creep rate values of asphalt under different aging styles are presented in Figure 4.

The results demonstrated that asphalt binder exhibited distinct variations in stiffness modulus and creep rate under different aging styles, with stiffness modulus increasing and creep rate decreasing to varying degrees. These changes reflected deteriorated low-temperature flexibility and stress relaxation capacity, ultimately leading to impaired low-temperature performance. Compared to PAV aging, both 12-month natural thermal-oxidative aging and all-weather aging induced significantly greater alterations in stiffness modulus and creep rate. For instance, at −12 °C, the stiffness modulus increased by 247.2% and 231.7% for natural thermal-oxidative aging and all-weather aging, respectively, relative to short-term aged asphalt binder. In contrast, PAV aging only caused a 61.6% increase in stiffness modulus under the same conditions. Compared to PAV aging, the stiffness modulus after natural thermal-oxidative aging and all-weather aging increased by an additional 114.8% and 105.3%, respectively. In cold-arid regions, extreme diurnal temperature fluctuations and prolonged daytime heat synergistically amplified aging rates, resulting in substantially accelerated degradation. Consequently, both natural aging modes produced far more severe low-temperature performance deterioration than PAV aging. Although PAV aging is widely considered equivalent to 3–10 years of natural aging [17], this equivalence depends critically on evaluation metrics, asphalt type, and climatic conditions. The complex aging conditions in cold-arid regions significantly accelerated the aging behavior of asphalt binder, thereby resulting in substantially inferior low-temperature performance of both naturally aged asphalt binder styles (thermal-oxidative and all-weather aging) compared to PAV-aged asphalt binders.

While asphalt binder in all-weather aging styles exhibited a higher stiffness modulus at −6 °C compared to natural thermal-oxidative aging, it demonstrated lower stiffness modulus at −12 °C and −18 °C. The creep rate of these also exhibited varying magnitude relationships at different temperatures, leading to multiple types of changes in the low-temperature performance of all-weather aged asphalt compared to natural thermal-oxidative aged asphalt. But on the whole, this change was not significant, thus confirming that thermal-oxidative effects remain the dominant factor driving asphalt aging. In reality, all-weather aging introduced additional environmental influences—such as light, water, and dust—absent in natural thermal-oxidative aging. Similarly, ultraviolet (UV) radiation synergized with oxygen to induce photo-oxidative aging of asphalt binder. Water exerted dual opposing effects: it leached aromatic hydrocarbons from asphalt through a washing action [35]. while simultaneously forming surface water films that inhibited photo-oxidative aging of asphalt binder [36]. Concurrently, dust accumulation partially shielded asphalt binder from UV exposure, further inhibited the aging speed of asphalt binder. These complex interactions among light, water, and dust under natural conditions created complex aging-induced behaviors, ultimately manifesting as intricate low-temperature performance deterioration behaviors in asphalt binder.

### 3.2. The Influence of Different Aging Styles on the Chemical Structure of Asphalt

#### 3.2.1. Chemical Structure Analysis

**Figure 5:**
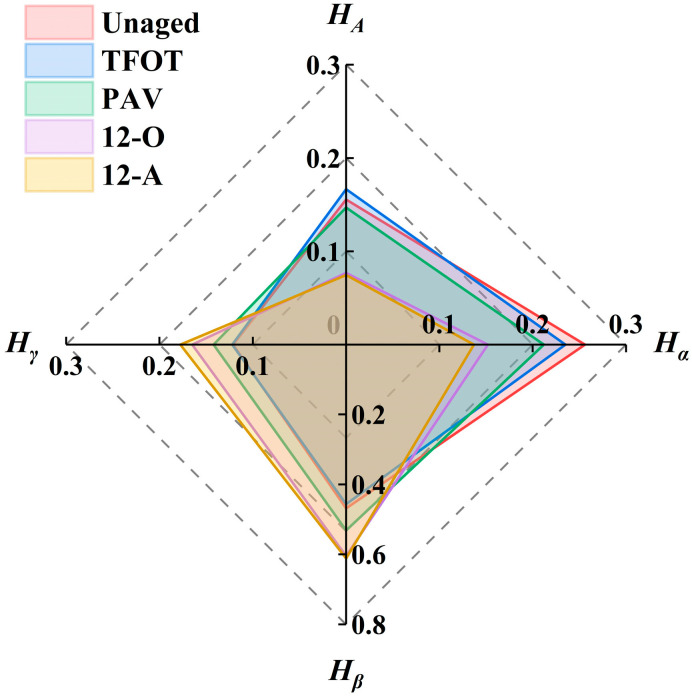
The attributed hydrogen content of asphalt binder under different aging styles.

Nuclear magnetic resonance (NMR) technology has been widely applied in the chemical structural analysis of asphalt binder and organic compounds, enabling precise determination of hydrogen atoms’ chemical environments in samples. Nuclear magnetic resonance (NMR) spectroscopy provides hydrogen-specific information, including chemical shifts, coupling constants, and integral values, which correspond to absorption positions, peak splitting patterns, and absorption intensities, respectively. These three spectral features enable the deduction of hydrogen atom positions along carbon chains, thereby determining the chemical structure of the analyzed sample. *H_A_* denotes hydrogen atoms directly bonded to aromatic carbons. *H_α_* denotes hydrogen attached to α-carbons adjacent to aromatic rings. *H_β_* denotes hydrogen on β-carbons of aromatic rings as well as CH_2_ and CH groups outside β-positions. *H_γ_* represents hydrogen on γ-carbons of aromatic rings and CH_3_ groups outside γ-positions. Through peak integration within the respective spectral regions of *H_A_*, *H_α_*, *H_β_*, and *H_γ_* followed by normalization, the contents of these four attributed hydrogen types were quantitatively determined, as illustrated in Figure 5.

The results demonstrated that after PAV aging, the *H_A_* and *H_α_* contents of asphalt binder exhibited certain reductions, indicating the occurrence of dehydrogenative condensation reactions on aromatic rings [20]. And the number of substituent groups on the aromatic rings of asphalt decreased. Furthermore, prolonged aging resulted in an increase in *H_β_* and *H_γ_* contents, indicating an accumulation of free chain structures within the asphalt binder. Following PAV aging, the *H_A_* and *H_α_* contents of asphalt binder decreased by 11.8% and 9.8%, respectively, compared to short-term aged asphalt binder, while *H_β_* and *H_γ_* contents increased by 16.4%. These results demonstrated that the chemical structure of asphalt undergoes more pronounced alterations under natural thermal-oxidative conditions, thereby significantly impacting its macroscopic performance. Following 12 months of all-weather aging, although minor increases or decreases in the four attributed hydrogen contents were observed compared to 12-month natural thermal-oxidative aging, these variations were not significant. This confirmed that thermal-oxidative aging is the main factor leading to the change in the chemical structure of asphalt binder.

#### 3.2.2. Elemental Content Analysis

**Figure 6:**
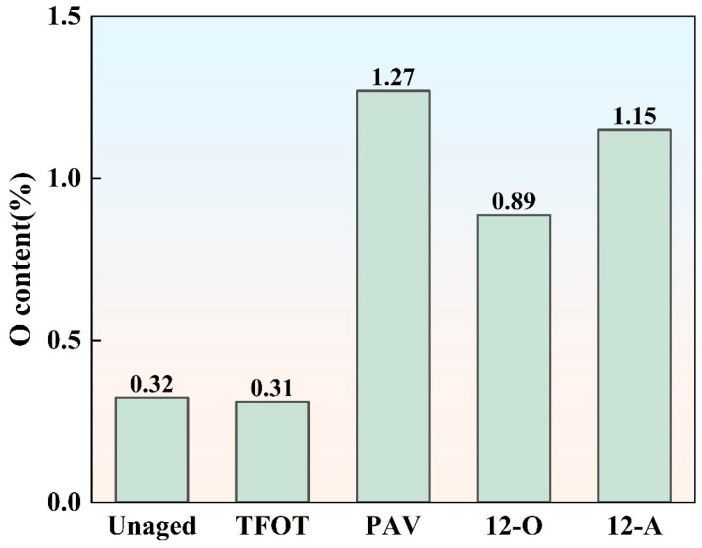
The content of *O* element in asphalt binder under different aging styles.

The aging process of asphalt binder is primarily driven by oxidation reactions. To further quantify the differences in oxidation reactions between natural aging styles and PAV aging, the oxygen content of asphalt binder under various aging styles were measured, as shown in Figure 6.

As shown in Figure 6, the oxygen content of asphalt binder significantly increased after long-term laboratory and natural aging. This rise was attributed to prolonged oxidation reactions that generate oxygen-containing groups within the asphalt binder. The asphalt binder subjected to PAV aging exhibited the highest oxygen content of 1.2%, demonstrating that more oxidation reactions occurred during the PAV aging process. After 12 months of natural thermal-oxidative aging, the oxygen content of asphalt binder was only 0.89%. Although the inclusion of light, water, and dust during 12-month all-weather aging increased the oxygen content compared to natural thermal-oxidative aging, it remained 9.4% lower than that of PAV-aged asphalt binder. This demonstrated that the high-temperature and high-pressure conditions of PAV aging significantly accelerate oxidation reactions in asphalt binder.

#### 3.2.3. Molecular Weight Analysis

**Figure 7:**
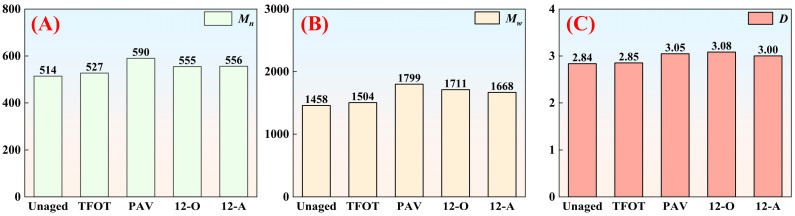
The molecular weight and distribution width of asphalt under different aging modes: (**A**) number-average molecular weight *M_n_*; (**B**) weight-average molecular weight *M_w_*; (**C**) polydispersity index *D*.

As a multicomponent composite material, asphalt binder consists of organic compounds with diverse molecular weights, resulting in a broad molecular weight distribution. During the aging process of asphalt binder, external environmental factors drive continuous chemical reactions among these compounds, generating new substances and altering the molecular weight profile of the asphalt. The molecular weight measurement results are shown in Figure 7.

As shown in Figure 7, both the number-average molecular weight and weight-average molecular weight of asphalt increased under all aging styles, with PAV-aged asphalt binder exhibiting the highest molecular weights. This phenomenon correlates strongly with the intensified oxidation reactions of asphalt binder induced by PAV aging. The oxidation reactions of asphalt binder generated oxygen-containing polar functional groups. Electrostatic interactions between these polar groups further promoted the aggregation of smaller molecules into larger macromolecular structures through association and cross-linking. Consistent with elemental analysis results, although natural aging caused more severe low-temperature performance degradation compared to PAV aging, the magnitude of molecular weight increase in naturally aged asphalt binder was significantly lower than that of PAV-aged asphalt binder.

The polydispersity coefficient could characterize molecular weight distribution. Compared with short-term aging, PAV aging, natural thermal-oxidative aging, and all-weather aging increased asphalt binder’s polydispersity coefficient by 6.8%, 8.1%, and 5.2%, respectively, indicating enhanced molecular dispersion within asphalt binder. A higher polydispersity coefficient corresponds to reduced dispersibility of maltenes, which deteriorates asphalt binder’s colloidal stability [37]; thereby this further deteriorated the low-temperature performance of asphalt binder.

### 3.3. Correlation Analysis of Macroscopic and Microscopic Performance of Asphalt Binder Under Different Aging Styles

**Figure 8:**
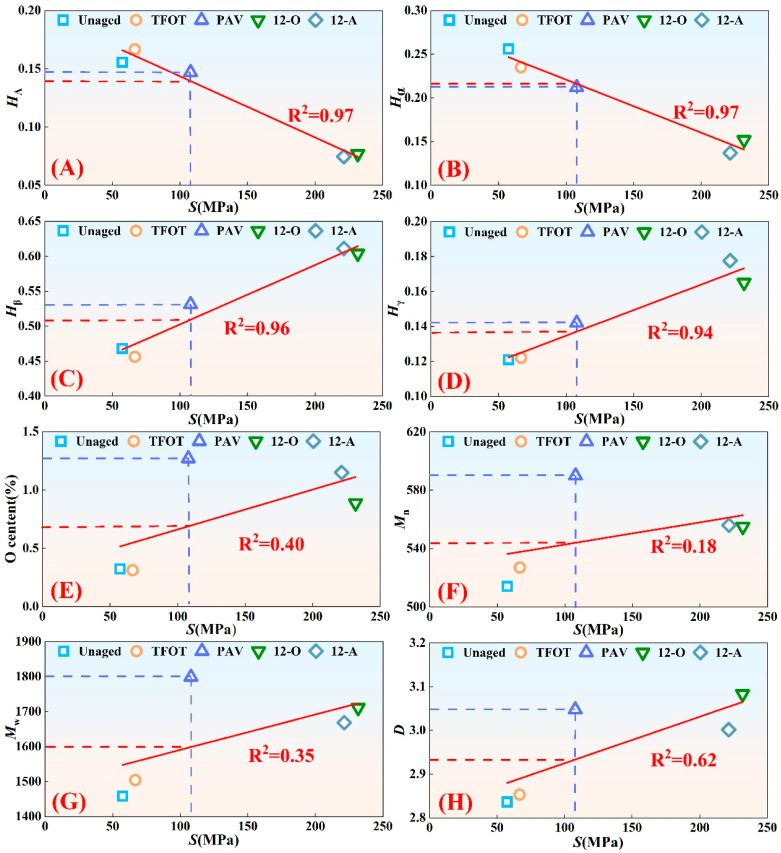
Correlation analysis of macroscopic and microscopic performance of asphalt binder under different aging styles: (**A**) Correlation between *H_A_* and *S*; (**B**) Correlation between *H_α_* and *S*; (**C**) Correlation between *H_β_* and *S*; (**D**) Correlation between *Hγ* and *S*; (**E**) Correlation between *O* content and *S*; (**F**) Correlation between *H_β_* and *S*; (**G**) Correlation between *H_β_* and *S.* (**H**) Correlation between *D* and *S*.

Previous studies demonstrate strong correlations between asphalt binder’s macroscopic performance and microscopic composition, indicating that cross-scale behaviors exist where microstructural changes predict macroscopic performance variations [38,39,40,41]. Given PAV aging’s forced aging characteristics, this forced aging process may modify asphalt binder’s cross-scale behaviors of macro-micro performance compared to natural aging. Therefore, correlation analyses between asphalt binder’s low-temperature performance and microscopic chemical composition were conducted across different aging styles. Low-temperature performance was quantified using the −12 °C stiffness modulus, as detailed in Figure 8.

As evidenced in Figure 8, although natural aging of asphalt binder involved exposure to light, water, dust, and other environmental factors, the inclusion of these factors did not significantly affect the cross-scale behaviors of its macro-micro performance. This phenomenon was likely attributable to the coupling effects of multiple factors, demonstrating that thermal-oxidative aging played a dominant role in governing the cross-scale behaviors of asphalt binder’s macro-micro performance during natural aging.

The stiffness modulus of asphalt binder under different aging styles exhibited strong linear correlations with four attributed hydrogen (*H_A_*, *H_α_*, *H_β_*, and *H_γ_*), with all goodness-of-fit (R^2^) values exceeding 0.94. It could be seen that after PAV aging and natural aging, the low-temperature performance of asphalt binder is affected by the changes of *H_A_*, *H_α_*, *H_β_*, and *H_γ_* in a similar trend. However, asphalt binder’s stiffness modulus under different aging styles showed weak linear correlations with oxygen content, number-average molecular weight, weight-average molecular weight, and polydispersity index, achieving a maximum goodness-of-fit of only 0.62. It could be seen from the figure that the diminished goodness-of-fit primarily stems from the PAV-aged asphalt binder data points significantly deviating the regression curve. This indicated that although laboratory PAV aging had resulted in higher oxygen content, number-average molecular weight, weight-average molecular weight, and polydispersity index of asphalt binder compared to natural aging, the laboratory PAV-aged asphalt binder did not exhibit a greater stiffness modulus. Instead, its stiffness modulus was markedly lower than that of naturally aged asphalt binder. These findings demonstrated that forced aging in the laboratory altered the cross-scale behaviors connecting oxygen content evolution, molecular weight changes, and low-temperature performance variations during asphalt aging.

Although research had established the predominant contribution of thermal-oxidative conditions to the cross-scale behaviors of asphalt binder’s macro-micro performance during natural aging, a thermal-oxygen-based PAV aging style still failed to restore authentic natural aging behaviors. Future research can achieve more precise aging simulations of asphalt binder by calibrating temperature and oxygen parameters in laboratory aging protocols.

## 4. Conclusions

In this investigation, testing was conducted on asphalt binder’s low-temperature performance and microscopic composition across unaged and four distinct aging styles. A preliminary analysis of macro-micro performance correlations was conducted, leading to four principal conclusions:

(1) The harsh climate of cold-arid regions accelerated the aging of asphalt binder. After natural thermal-oxidative aging and all-weather aging, the stiffness modulus of asphalt at −12 °C increased by 247.2% and 231.7%, respectively, significantly exceeding the 61.6% increase observed after PAV aging. This poses greater challenges to the durability of asphalt pavements in cold and arid regions.

(2) Natural aging produced more pronounced changes in four attributed hydrogen content than PAV aging, whereas oxygen content and molecular weight variations were less. The results demonstrated that dehydrogenative condensation and chain scission reactions were not prominently observed during the PAV forced aging process. However, significant oxidation occurred, leading to the formation of more macromolecular structures in the asphalt.

(3) From both macroscopic and microscopic perspectives, thermal-oxidative conditions were identified as the primary cause of asphalt binder aging in natural environments. Environmental factors including light, water, and dust contamination exhibited either accelerating or inhibitory effects on aging processes. The coupling of these multiple factors did not significantly alter the aging pathway of the asphalt binder.

(4) The poor correlation (with all R^2^ values below 0.62) between oxygen content, molecular weight, and low-temperature performance under various aging modes was primarily due to the deviating data points corresponding to PAV-aged asphalt binder. This demonstrated that the forced aging of PAV aging fundamentally modified the asphalt binder’s cross-scale behaviors of linking microscopic component composition to macroscopic performance.

This study preliminarily investigated the differences in aging pathways between laboratory-accelerated and natural aging of asphalt binder. The findings indicate that variations in thermal-oxidative conditions serve as the primary factor contributing to these distinct aging paths. This research has established the fundamental causes underlying the divergent aging pathways between laboratory and field conditions, thereby providing data support for the design of more accurate accelerated aging tests for asphalt binder. In future research, consideration should be given to increasing aging duration and incorporating additional aging methods to achieve alignment between laboratory-accelerated and natural aging pathways of asphalt binder.

## Figures and Tables

**Table 1:** Basic physical indexes of virgin asphalt binder.

Tests		Units	Results	Limits	Standards
Penetration (25 °C, 100 g, 5 s)		0.1 mm	81.5	80~100	T0604-2011
Softening point (R&B)		°C	45	≥44	T0606-2011
Ductility (5 cm/min, 10 °C)		cm	100	≥30	T0605-2011
Aging tests (163 °C, 5 h)	The quality change	%	−0.13	≤±0.4	T0610-2011
	Penetration ratio	%	64	≥57	T0604-2011
	Residual ductility ratio	%	11	≥8	T0605-2011

## Data Availability

The original contributions presented in the study are included in the article, further inquiries can be directed to the corresponding author.
